# Quantitative PCR as an Alternative in the Diagnosis of Long-QT Syndrome

**DOI:** 10.1155/2013/418604

**Published:** 2013-07-02

**Authors:** Ewa Moric-Janiszewska, Ludmiła Węglarz, Magdalena Szczurko

**Affiliations:** Department of Biochemistry, Medical University of Silesia, Narcyzów 1, 41-200 Sosnowiec, Poland

## Abstract

Congenital long-QT syndrome is a genetic disorder associated with abnormalities in the function and/or structure of cardiac ion channels. Up to the present, 13 types of the disease have been described (LQTS1-13) which result from the fact that 13 genes of which mutations can have an influence on the occurrence of the disease have been identified. Characteristic symptoms of the disease include the changes in the ECG (QT interval prolonged above 450 ms), “*torsade de pointes,*” fainting, and even sudden cardiac death. The present study has been focused on two types of the disease, namely, LQTS1 and LQTS2. The examination of two appropriate genes expression (*KCNQ1; KCNH2*) at the transcription level by QRT-PCR in a group of LQTS patients and a healthy control group showed different transcriptional activities of *KCNH2* gene in LQTS2 patients compared to the control individuals. *KCNQ1* gene expression study did not reveal such differences between both groups. The results indicate that QRT-PCR may serve as a complimentary method to the identification of molecular alterations in genetic determinants of LQTS2 only, but it cannot be used as a sole diagnostic criterion.

## 1. Introduction 

Long-QTS (long-QT syndrome) is a hereditary disorder associated with myocyte ion channel abnormalities. The disease is characterized by prolonged QT interval and unique morphology of the T wave on ECG. The syndrome is also manifested by the occurrence of *“torsade de pointes”* (TdP). Prolonged episodes of TdP may turn into a ventricular fibrillation (VF) [1, 2]. The affected patients may show many other accompanying symptoms such as palpitations, fainting, dizziness, paleness, weakness, heart rhythm disturbances, and, finally, sudden cardiac death (SCD) [1, 3–5].

Until recently, the mutations in the thirteen genes have been identified to be genetic causes of  long-QT syndrome [6–10]. Most of them (about 75%) have been found in *KCNQ1*, a gene encoding the I_Ks_ subunit of the slow potassium channel, *KCNH2 (HERG)*, that encodes the I_Kr_ subunit of the rapid potassium channel and *SCN5A* encoding subunit of the sodium channel [11] (Table 1).

Real-time QRT-PCR (Real-Time Quantitative Reverse Transcriptase Polymerase Chain Reaction) is a common test tube method used for amplifying a selected DNA sequence that is used in genetic testing. It is a modification of the classical polymerase chain reaction (PCR). The method includes reverse transcriptase, an enzyme that uses RNA as a template to rewrite it into cDNA (a strand of DNA complementary to mRNA) [12–15]. This method requires detection of the amplified PCR products, specialized thermocycler cooperating with the fluorimeter for continuous fluorescence monitoring during the reaction, [13] and specialized software for quantitative analysis [12]. Specific fluorescent-labeled probes or nonspecific fluorescent dyes are used to monitor the level of amplification and the amount of product.

The overarching aim of the study was to demonstrate whether genetic testing performed by QRT-PCR technique can be used as an alternative approach in the diagnosis of the long-QT syndrome and whether QRT-PCR is a suitable diagnostic method for determining the number of mRNA transcripts of genes responsible for types 1 and 2 of long-QT syndrome manifestation.

Determination of the number of mRNA copies of *KCNQ1* (LQTS1) and *KCNH2* (LQTS2) was supposed to confirm the presence of the long-QT syndrome in the group of patients from the families in whom the syndrome has been previously diagnosed on the basis of supraventricular arrhythmia by ECG.

Two research hypotheses were formulated and tested in this study.

1. The number of *KCNQ1* mRNA copies (expression) in patients diagnosed with type 1 LQT syndrome is different in comparison to the healthy individuals.
2. The number of *KCNH2* (*HERG*) mRNA copies (expression) in patients diagnosed with type 2 LQT syndrome is different compared to the healthy individuals.

## 2. Material and Methods 

The studies included six families whose 34 members have been examined for the long-QT syndrome at the Department of Pediatric Cardiology, Medical University of Silesia in Katowice-Ligota. Types and subtypes of the disease were diagnosed with the use of clinical tests, including ECG.

**Figure 1:**
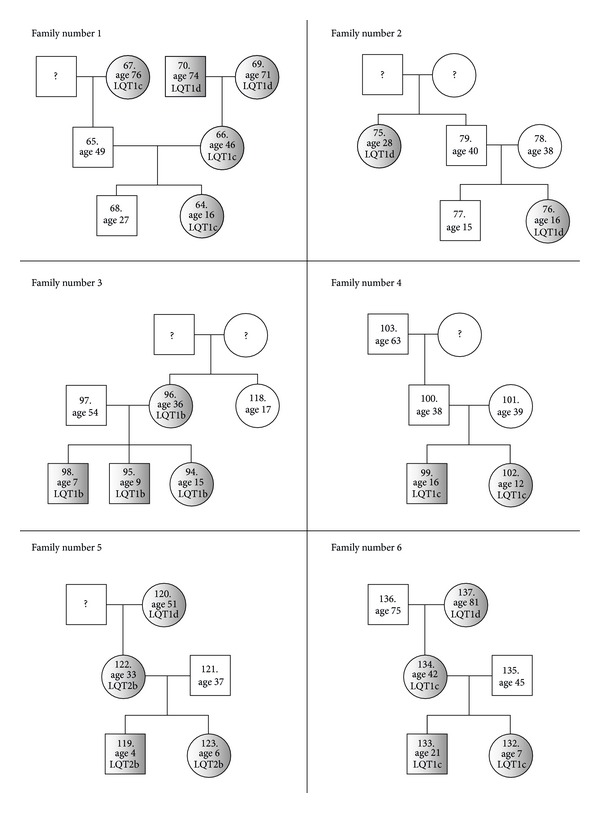
Families 1–6 genealogy. Circle is the equivalent of the female sex, and square is the equivalent of the male sex. Each family member is described with the sample number, age at the time of the study, and the type and the subtype of the disease (for patients diagnosed with LQTS). Grey color indicates patient diagnosed with LQTS, and white color indicates healthy individuals. The question mark represents a person who was not examined.

The long-QT syndrome was recognized in 21 individuals, 18 of whom were diagnosed with type 1 LQTS (LQTS1b—4 individuals, LQTS1c—8 individuals, LQTS1d—6 individuals) and the other 3 with type 2 LQTS (LQTS2b). Thirteen members of the families showed no clinical symptoms of the disease and they were healthy (Figure 1).

The consent to use blood samples taken from patients was obtained from the Bioethics Commission of the Medical University of Silesia in Katowice. 

The experimental group was set up based on specific inclusion and exclusion criteria. The inclusion criteria involved the first-degree relatives with known long-QT syndrome (based on diagnostic criteria by Schwarz and Moss >1p).

The exclusion criteria for healthy individuals included enrollment of all patients according to the scale of Schwarz and Moss <1p and outside the sinus rhythm, patients with AV block or right bundle branch block with sick sinus syndrome, and structural heart disease and inflammation of the heart muscle, patients with electrolyte disturbances (F, Ca, Mg), patients with endocrine disorders and neurological diseases, and patients during drug therapies that may affect the repolarization period.

There has been phenotypic classification of patients with long-QT syndrome within the two most common types of long-QT syndrome, LQTS1 and LQTS2, that is, having intelligence and are different compared to control durations of individual parameters repolarization QT QTo, QTP, and TpTe and morphological evaluation of the T wave in the ECG standard and stress. Division of LQTS subtypes was made based on the morphology of repolarization (QT period) and the morphology of the T wave as Ia: “infant type” of T-wave, Ib: T wave with a characteristic wide base, Ic: proper morphology of the T wave, Id: the late beginning of the T wave, IIa:two-humped T wave, IIb: T wave slightly two-humped in the peak region, and IIc: T wave slightly two-humped in the descending portion.

As a material for the study, RNA isolated from peripheral blood mononuclear cells was used (Fenozol total RNA isolation Reagent Set A&A Biotechnology). Qualitative evaluation of the extracts was made via agarose gel electrophoresis (1%) with the addition of ethidium bromide. Quantitative assessment of  RNA extracts was performed with the use of the Gene Quant Pro spectrophotometer produced by Amersham Biosiences.

Transcriptional activity of the examined genes was determined using a commercially available kit (TaqMan Gene Expression Assays, Applied Biosystems, Foster City, CA, USA). The number of *KCNQ1* and *KCNH2* mRNA molecules was determined based on QRT-PCR reaction kinetics using ABI  PRISM  7000 sequence detection system (Applied Biosystems) and a kit containing fluorescent dye (ROX QuantiTect Probe RT-PCR, Quiagen, Germany).

QRT-PCR was performed in one step using a reaction mix containing 25 *μ*L 2x QuantiTect Probe RT-PCR Master Mix (HotStarTaq DNA Polymerase, QuantiTect Probe RT-PCR buffer containing Tris-HCl, KCl, (NH_4_)_2_SO_4_, 8 mM MgCl_2_, pH = 8.7, dNTP mix, ROX reference dye), 0.5 *μ*L QuantiTect RT Mix (Omniscript Reverse Transcriptase, Sensiscript Reverse Transcriptase in commercially available concentrations), and 1 *μ*L of starters and T Gene Expression Assay probes (Applied Biosystems), RNA template, and apyrogenic water. Together with the examined genes, commercially available DNA standard (**β*-actin*, Applied Biosystems) was amplified as internal control in each single QRT-PCR for all samples.

Reverse transcription reaction was performed at 50°C for 30 min. Following initial activation of HotStar Taq DNA Polymerase at 95°C for 15 min, a two-stage reaction was carried out with denaturation at 94°C for 15 sec and starter ligation at 60°C for 60 sec. Final elongation of amplificated products was achieved at 72°C for 10 min.

## 3. Statistical Analysis

The expression level of the examined genes was inferred on the basis of number of their mRNA copies per 1 *μ*g of total RNA. Statistical significance of *KCNQ1* and *KCNH2* expression values obtained for experimental group was measured using Mann-Whitney *U* test. The results were judged significant if *P* < 0.05. 

## 4. Results 

Examined families are shown in Figure 1.

The expression of genes* KCNQ1* (LQTS1), *KCNH2 *(*HERG*-LQTS2), and **β*-actin* was analyzed for patients diagnosed with LQTS and healthy individuals (Table 2).

### 4.1. The Comparison of the Gene Expression Depending on Gender and Subtype of LQTS

**Figure 2:**
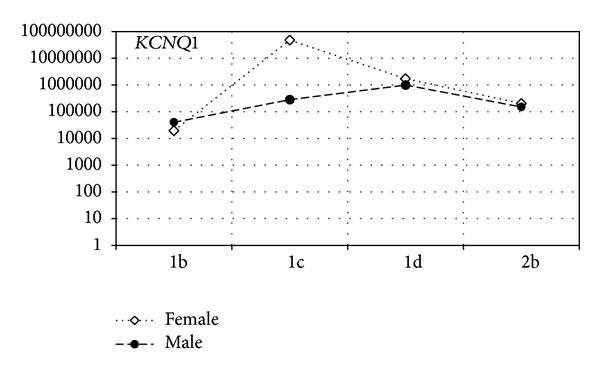
The ratio of mRNA copy number for *KCNQ1* in the female and male groups of LQTS patients. The *x*-axis shows type and subtype of LQTS, and the *y*-axis shows the average values of mRNA copies of the genes.

**Figure 3:**
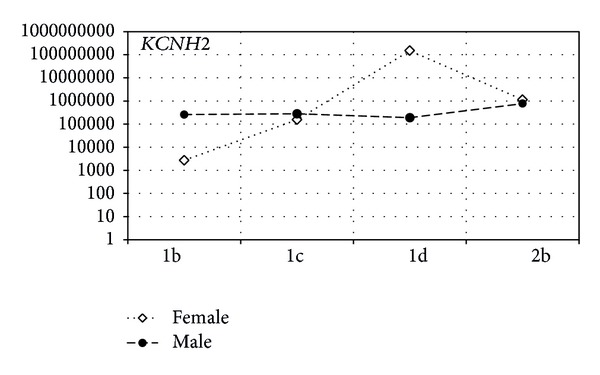
The ratio of mRNA copy number for *KCNH2* in the female and male groups of LQTS patients. The *x*-axis shows type and subtype of LQTS, and the *y*-axis shows the average values of mRNA copies of the genes.

Figures 2 and 3 show a statistical trend in the difference of gene expression of tested genes in respect of gender and subtype of LQTS.

As presented in Figure 2, the highest expression of *KCNQ1* gene was observed in females compared to males. The highest level of mRNA copies of *KCNQ1* gene has been reported in patients with diagnosed subtype LQTS1c and the lowest in patients with LQTS1b subtype of the disease.

As presented in Figure 3, the highest expression of *KCNH2 *gene was observed in female patients compared to males. The highest level of mRNA copies of *KCNH2* gene has been reported in patients with diagnosed subtype LQTS1d and the lowest in patients with LQTS1b subtype of the disease also.

## 5. Discussion

The level of knowledge in the field of inherited heart diseases has significantly been increasing due to dynamic development of genetics. For some channelopathies and cardiomyopathies, a direct cause of a genetic disorder was found in a large percentage of the study groups with the use of genetic tests. As to the long-QT syndrome, the genetic defect that is responsible for the disease can be identified in up to 70% of patients undergoing genetic testing. In case of genetic diseases, a variety of results, that is, positive ones indicating a mutation that is certainly responsible for the disease occurrence, mutations of unproven clinical significance or mild mutations (VUS—variant of unknown significance), which are called “genetic noise”, can be obtained using genetic tests. For LQTS patients the type of  “genetic noise” is detected on the level of 4% by genetic testing [16, 17].

Recent literature reports on the level of knowledge and application of genetic tests for inherited heart diseases diagnosis have been published in a novel publication of HRS and EHRA experts [16, 18]. However, genetic tests are not helpful in all cases and must be confronted with the results of clinical trials, clinical symptoms, and a family history for each patient individually and should not be considered as the sole diagnostic method [16, 19].

Mutations in 13 different genes are responsible for the congenital, genetically determined LQT syndrome, and on this basis, 13 types of the disease (LQTS1-13) were described. Mutations in those genes encoding ion channel proteins (usually sodium and potassium) located in the myocardium, result in their structural abnormalities and/or loss of function and incorrect cardiac repolarization [16, 20–23].

The analysis was intended to verify whether the use of genetic test—QRT-PCR—carries a diagnostic weight for detecting two major types of disease LQTS 1 and 2.

In humans *KCNQ1* gene is expressed in pancreas, kidneys, placenta, and lungs. The highest mRNA transcript levels of *KCNQ1* gene were observed in heart muscle cells; no expression was found in brain, skeletal muscles, or liver. The highest mRNA transcript levels of *KCNH2* gene were observed in heart muscle and brain [7]. In the diagnosis of LQTS, as well as in the risk analysis, a lot of aspects such as the measurement of the QT interval, sex, age, clinical characteristics (loss of consciousness, sudden cardiac events, etc.), and genotype, are taken into account.

In this study, the members of six families clinically diagnosed with LQT1 or LQT2 syndrome were examined. The results of analysis confirmed one of the hypotheses indicating the presence of a statistical trend that the number of *KCNH2* mRNA copies is higher in patients with LQTS2 than in healthy individuals (Mann-Whitney *U* test *P* = 0.014).

Overexpression of *HERG* is known to have implications in the construction of the ion channel. The protein product of *HERG* forms *α* subunit of rapid potassium channel (I_Kr_) which is responsible for the final phase of repolarization in cardiomyocytes and for the prevention of premature myocardial arousal. As a result of increased *HERG* copy number, an intensified potassium channel protein translation in patients occurs, which results in an improper construction of I_Kr_ channel (too much protein creating *α* subunit). In view of the presence on the N-terminal fragment of the protein, which is encoded by this gene, a conservative PAS region (Per-Arnt-Sim), its overexpression may have a negative effect. Since the role of the PAS region is to regulate channel inactivation process, the altered amount of a protein can have a negative influence on channel's function [24, 25].

The hypothesis concerning different *KCNQ1* gene expressions in people diagnosed with LQTS1 compared to healthy individuals was not statistically confirmed, probably due to the study limitations.

Our former study on age- and sex-dependent level of expression of *KCNQ1* and *KCNH2* gene with the use of QRT-PCR method was one of the first which used human whole blood samples. The study showed age- and sex-dependent differences in the level of expression of both genes. Healthy women were characterized by a much lower number of *KCNQ1* and *KCNH2* mRNA copies in comparison to healthy male subjects. In addition, healthy adults showed a much higher number of *KCNQ1* mRNA copies than healthy children, while healthy children had significantly lower *KCNH2* mRNA copy number in comparison to healthy adults. The level of *KCNQ1* and *KCNH2* expression was also compared in a group of adult patients under and over 55 years of age. Among adult patients under 55 years of age a very high number of *KCNQ1* mRNA copies and underestimated number of *KCNH2* mRNA copies were demonstrated compared to patients above 55 years of age [7].

In the present study, the level of *KCNQ1* and *KCNH2* expression in the group of female patients relative to the group of male patients was analyzed. Comparative analysis of the average mRNA copy number of both genes showed much higher number of *KCNQ1* and *KCNH2* mRNA transcripts in women than in men with the disease.

A lot of factors are believed to influence the tendency to cardiac arrhythmias occurrence and QT prolongation on the ECG in women. One of them is the very low level of androgens in relation to men. It is known that androgens have a protective effect on the heart and help to shorten the QT interval. Another causative factor may be genetically determined difference in the density of potassium channels located in the cell membrane of cardiomyocytes. Estrogen effects on the kinetics and the function of potassium and calcium channels and regulation of ion channels expression as well as the dominance of the parasympathetic system also are not without significance. The differences in the length of the QT interval are also caused by lower myocardial weight in women compared to men, different chest configuration, and the different ionic structure of the cell membrane in cardiomyocytes. Age and gender have a significant impact on the clinical course of the disease. It has been shown that the risk of dangerous cardiac events is increased in boys before adolescence, while it is increased in women in adolescence and later [7, 26, 27].

Another study on *KCNQ1* and *KCNH2* expression in patients diagnosed with LQTS2 or LQTS1 and in healthy subjects with the use of QRT-PCR method showed that the length of the QT interval in LQTS1 patients was independent on the gene expression level. However, a relationship between the amount of *KCNQ1* mRNA copy number and the length of the QT interval in LQTS2 patients was found. The correlation showed that the lower *KCNQ1* mRNA copy number was, the longer QT interval in patients with type 2 LQTS was observed. Surprisingly, in the case of *KCNH2* gene of which mutation affects type 2 of LQTS, the expression level of the gene showed no effect on QT interval in patients with LQT2. This result could possibly be caused by an insufficient number of both healthy and sick individuals in the study group [3]. Based on the this study, it can be said that higher *KCNQ1* expression level in LQTS1 patients may be an additional distinctive criterion in the diagnosis of LQTS1. Moreover, it was concluded that it is not possible to confirm or exclude the presence of LQTS2 on a basis of *KCNH2* expression. Furthermore **β*-actin* has been shown to be a proper endogenous control in the analysis of *KCNQ1* and *KCNH2* expression level in patients with LQTS [3].

The above-described results of our study [3] do not coincide with the results presented in the current study, which revealed that individuals with LQTS2 exhibited increased *KCNH2* expression (expressed in mRNA copy number) compared to healthy individuals and that on a basis of *KCNQ1* expression profile analysis it is not possible to confirm or exclude the presence LQTS1. Certainly, this issue requires further study. The present study also confirmed that **β*-actin* control gene was properly chosen, as there was no statistically significant difference *P* = 0.620, *P* = 0.624 (Mann-Whitney *U* test) in its expression between both groups.

In this study the relationship between *KCNQ1* and *KCNH2* mRNA copy number and the type and subtype of the disease have been analyzed for the first time. The analysis showed the relationship between the amount of mRNA copies of of *KCNQ1* gene and the clinical type of the disease as follows: LQTS1c > LQTS1d > LQTS2b > LQTS1b. The lowest average transcript molecules number was observed in the group of LQTS1b patients, while its highest number was in patients with LQTS1c. For *KCNH2* gene the relationship between the amount of mRNA copies and the clinical type of the disease was as follows: LQTS1d > LQTS2b > LQTS1c > LQTS1b. The lowest expression level of this gene was observed in the group of LQTS1b patients, while the highest was in patients with LQTS1d. Data concerning such relationship analysis have not been published until now.

The results of this study also indicate different levels of gene expression among studied families. *KCNQ1* expression comparison in 1–6 families revealed the highest average mRNA copy number in the LQTS1 families 1 (66,979,159 copies) and 2 (67,860,833 copies). The lowest average mRNA copy number of this gene was found in LQTS1 family 4 (37,921 copies). *KCNH2* expression analysis in 1–6 families indicated the highest average mRNA copy number in family 2 (545,217,632 copies) and the lowest expression of this gene in family 1 (50,110 copies). In family 5, the only family, diagnosed with LQTS2 the transcriptional activity of this gene was low (644,017 copies).

These results may be explained by the existence of microRNA (miRNA), which in many conditions can have an influence on the occurrence of arrhythmias or an important role in the context of myocardial excitability through the regulation of ion channels, transporters, and cellular protein. A single miRNA can modulate the expression of many different genes with similar functions and affect the phenotype and disease activity. The excess of miRNA may also play a role in a paradoxical increase of target gene expression [28, 29].

Moreover, epigenetic mechanisms of gene regulation may cause many single-gene, as well as poly-gene, genetic diseases. Epigenetics is one of the fastest growing fields in studies of genetic variation which are independent on the primary sequence of  DNA but is a result of specific regulatory mechanisms activity, such as DNA methylation, modification of histone proteins, and the expression of antisense RNA or interference RNA. Phenotype diversity of LQTS may suggest epigenetic modifications of the corresponding genes [7, 30].

In 2007, Miller et al. [31] decided to establish peripheral whole blood to be a suitable material for isolation of mRNA for genetic testing use in the diagnosis of certain diseases of the cardiovascular system, including LQTS. Their innovative results showed that the expression profile of genes, of which mutations are responsible for the occurrence of LQTS, can be successfully examined with the use of mRNA isolated from peripheral blood.

Until now, the use of mRNA for genetic tests was recommended only in the case of searching the mutations in large genes. mRNA used for genetic testing was derived from the purified lymphocytes (collected from max. 50 mL of blood), skin biopsy, or fibroblasts culture. In addition, there was another limitation to the use of mRNA in the diagnosis of LQTS, namely, the fact that the expression of genes related to the occurrence of this disease is strictly limited to the myocardial cells and that mRNA transcripts could not be successfully extracted from lymphocytes or fibroblasts culture [31, 32].

Using observations from the microarray analysis, Miller et al. noted that the quaternary ammonium surfactants could greatly enhance the yield of rare peripheral blood mRNAs and increase the detection of tissue-restricted gene transcripts. The researchers proved that the cardiac restricted mRNA transcripts can be successfully detected in the peripheral blood without additional procedures required with cell culture, and so forth. Transcripts isolated from the whole blood can be used in the molecular methods [31].

Researches from the Medical University of Silesia, involved in the study of molecular diagnosis of LQTS families, have shown that due to the fact that gene expression is regulated by a number of nonlinear interactions between proteins, RNA and DNA and metabolites derived from different pathways. Due to the facts mentioned above the tested QRT-PCR method is limited in some extent [8].

First of all, the assessment of mRNA copy number of studied genes using QRT-PCR technology can determine the mRNA expression level (gene copy number/protein *μ*g). This method can only suggest possible changes in the number and/or function of the protein; however, the mRNA expression may not be correlated with the protein concentration [8]. Despite the relationships between gene expression and protein function, further analysis, such as analysis of proteins, will be needed to clarify the influence of *KCNQ1* and *KCNH2* mRNA copy number on the etiology of LQTS. Some limitation of QRT-PCR technique is also an appropriate level of transcription of the tested gene in the available tissue samples. The optimal material for such analysis could be a myocardium specimen. Unfortunately, myocardial biopsy is not routinely used in the diagnosis of LQTS, and it carries a high risk of complications [8].

It is widely recognized that *KCNQ1* and *KCNH2* expression is particularly restricted to the heart muscle and that mRNA transcripts of these genes cannot be isolated from lymphocyte cultures. However, Miller and colleagues [31] have overthrown this theory, showing that cardiac-specific mRNA can be detected in the peripheral blood [31].

The aim of the present study was to analyze the relationship of *KCNQ1* and *KCNH2* expression level with the occurrence of the long-QT syndrome. The study was based on the above-mentioned innovative research results indicating human peripheral blood to be convenient and suitable material for the study of LQT-related genes. It was found that the clear assessment of the influence of gene expression levels on the LQTS occurrence is not possible. In order to obtain a scrupulous analysis of this issue, a detailed research on a larger group of people must be performed to reduce the statistical error. Also the duration of blood storage time, the presence of thrombus, and the degree of total RNA extracts purification may influence the final result of the experiment.

In further work on this issue, cardiac biopsy specimens derived from patients clinically diagnosed with LQTS (study group) and from healthy individuals (control group) for the analysis of *KCNQ1* and *KCNH2* expression should be considered. In a view of the relationship between the myocardium ion channels function and the occurrence of LQTS, the myocardial tissue specimens may provide the best material to study the expression profile of these genes. However, due to invasiveness of endomyocardial biopsy and the high risk of complications, some studies using for these purposes peripheral blood are carried out [32].

## 6. Conclusions

1. The level of gene expression is different within studied families and specific subtypes of the disease.
2. Two types of the disease (LQTS1 and LQTS2) cannot be clearly differentiated using Taq-Man QRT-PCR method.
3. The results can be useful in the next steps of *KCNQ1* and *KCNH2* expression studies.
4. In subsequent work related to this research issue an increase of the study groups size should be considered, as well as the impact of blood storage time, the presence of thrombus, and the degree of total RNA extracts purification on the final result of the experiment.

## Figures and Tables

**Table 1:** The frequency occurrence of LQTS1-3, ECG characteristics for each type of the disease, and the most common stimuli leading to fatal cardiac events.

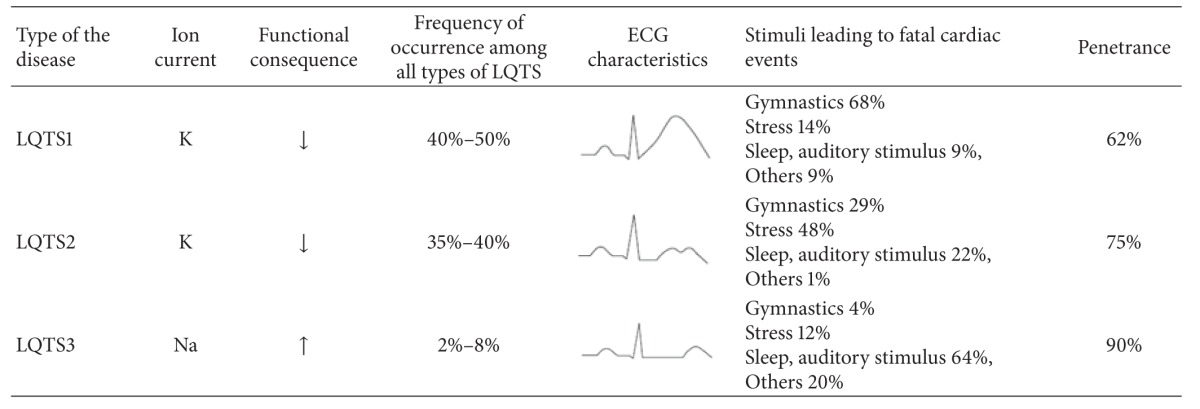

**Table 2:** Descriptive statistics of *KCNQ1*, *KCNH2*, and **β*-actin* mRNA copy numbers in the group of 21 patients diagnosed with LQTS and in the group of 13 individuals with no clinical symptoms of the disease.

		*KCNQ1*/LQTS1	*KCNH2*/LQTS2	*β-Actin *
Healthy individuals (*n* = 13)	Average	85148	78078	57078
	SD	39777014	146116043	10727502
	Median	65634041	496551188	35933549
LQTS patients (*n* = 21)	Average	212561	156930	27673
	SD	14035130	39665878	259808
	Median	33829344	166511950	566463
Mann-Whitney *U* test		*P* < 0.892	*P* < 0.014	*P* < 0.620
				*P* < 0.624
